# A Personalized Rituximab Retreatment Approach Based on Clinical and B-Cell Biomarkers in ANCA-Associated Vasculitis

**DOI:** 10.3389/fimmu.2021.803175

**Published:** 2022-01-12

**Authors:** Jack Arnold, Edward M. Vital, Shouvik Dass, Aamir Aslam, Andy C. Rawstron, Sinisa Savic, Paul Emery, Md Yuzaiful Md Yusof

**Affiliations:** ^1^ Leeds Institute of Rheumatic and Musculoskeletal Medicine, University of Leeds, Chapel Allerton Hospital, Leeds, United Kingdom; ^2^ National Institute for Health Research (NIHR) Leeds Biomedical Research Centre, Leeds Teaching Hospitals NHS Trust, Leeds, United Kingdom; ^3^ Haematological Malignancy Diagnostic Service, Leeds Teaching Hospitals NHS Trust, Leeds, United Kingdom

**Keywords:** B cell, rituximab, cyclophosphamide, immunoglobulin, vasculitis

## Abstract

**Background:**

Time to relapse after rituximab for the treatment of antineutrophil cytoplasmic antibody (ANCA)-associated vasculitis (AAV) is variable, and optimal retreatment strategy has remained unclear. In AAV following rituximab induction, the study objective was to evaluate clinical and B-cell predictors of relapse in order to develop a retreatment algorithm.

**Methods:**

A retrospective observational study was conducted in 70 rituximab-treated ANCA-associated vasculitis patients followed up for over 10 years. Complete response (CR) was defined as Birmingham Vasculitis Activity Score v3.0 = 0. Retreatment was given on clinical relapse, defined as new features or worsening of persistent disease (not by biomarker status). Peripheral B-cell subsets were measured using highly sensitive flow cytometry. Predictors were tested using multivariable Cox regression.

**Results:**

Median time to retreatment for cycles 1–5 were 84, 73, 67, 60, and 73 weeks. Over 467 patient-years follow-up, 158 relapses occurred in 60 patients; 16 (in 15 patients) were major (renal = 7, neurological = 4, ENT = 3, and respiratory = 2). The major-relapse rate was 3.4/100 patient-years. In multivariable analysis, concomitant immunosuppressant [HR, 0.48 (95% CI, 0.24–0.94)], achieving CR [0.24 (0.12–0.50)], and naïve B-cell repopulation at 6 months [0.43 (0.22–0.84)] were associated with longer time to relapse. Personalized retreatment using these three predictors in this cohort would have avoided an unnecessary fixed retreatment in 24% of patients. Area under the receiver operating characteristic for prediction of time to relapse was greater if guided by naïve B-cell repopulation than if previously evaluated ANCA and/or CD19^+^ cells return at 6 months had been used, 0.82 and 0.53, respectively.

**Conclusion:**

Our findings suggest that all patients should be coprescribed oral immunosuppressant. Those with incomplete response or with absent naïve B cells should be retreated at 6 months. Patients with complete response and naïve repopulation should not receive fixed retreatment. This algorithm could reduce unnecessary retreatment and warrant investigation in clinical trials.

## Introduction

Rituximab, a chimeric anti-CD20 monoclonal antibody is licensed for remission induction of antineutrophil cytoplasmic antibody (ANCA)-associated vasculitis (AAV). However, the majority of patients with AAV experience a clinical relapse following this initial induction and repeat cycles of rituximab are required for maintenance of remission (1–6). There is a need to establish an optimal long-term strategy that is effective and safe for rituximab-treated patients in AAV.

Three strategies have been proposed. (i) Fixed retreatment, which may vary internationally either using 500 mg × 2 infusions followed by 500 mg infusion every 6 months or 1,000 mg infusion every 4 months or 1,000 mg infusion every 6 months for 18 months (7, 8), with this regimen extended to 5 years in patients at higher risk of relapse (9). This is associated with low rates of relapse but may lead to hypogammaglobulinemia and serious infection; an effect that we showed was exacerbated if cyclophosphamide had also been previously used and which predicted severe infection (2, 10–13). (ii) Retreatment-on-clinical relapse. We have used this strategy and demonstrated low rates of hypogammaglobuliemia and a longer time to relapse of between 6 months and 4 years. However, this may permit severe disease flares and consequent glucocorticoid exposure (3). (iii) Retreatment according to biomarkers. This aims to avoid both problems by retreating according to predicted time to relapse.

Biomarker-led retreatment was investigated in the MAINRITSAN2 study using CD19^+^ cells or ANCA to trigger repeat cycles. However, this biomarker-led protocol resulted in numerically more relapses compared with fixed retreatment, 14/81 and 8/81 patients, respectively, but this difference was described as not statistically significant (14). Surprisingly, 11/19 (58%) patients with no B-cell return experienced ≥1 relapse while only 11/142 (8%) patients with B cells detected on at least one occasion had relapsed (*p* < 0.001 in *post-hoc* analysis).

The MAINRITSAN2 biomarker-led protocol used CD19^+^ cells as a pharmacodynamic and pharmacokinetic marker to guide an intention for perpetual absence of B cells. However, the association between CD19^+^ return and clinical relapse in this trial was indeed counterintuitive. More recent data have given a more nuanced picture of B-cell monitoring that explains the results from that trial. Analysis of B-cell subsets reveals disease-specific signatures. Systemic lupus erythematosus (SLE), for example, is characterized by expansion of plasmablast numbers in proportion to autoantibody repertoire (15, 16), while in contrast, AAV is characterized by naïve lymphopenia in proportion to CRP (3). In both these diseases, we showed that analysis of B-cell subsets in early repopulation after rituximab using highly sensitive flow cytometry (HSFC) can identify these signatures and guide retreatment decisions. Accordingly, in SLE, early plasmablast repopulation predicts early relapse. In AAV, repopulation of naïve B cells (which are the majority of cells detected by a CD19^+^ assay), is in fact a *good* prognostic marker for sustained response. Whereas failure to repopulate naïve B cells at 6 months is a sign of disease-specific B-cell activity and heralds early relapse (3).

Since our original publication (3), we have gathered data in more rituximab-treated patients with longer follow-up. Retreatment continued to be prescribed according to clinical relapse, enabling us to further evaluate clinical and B-cell relapse predictors. The objectives of the present study were to validate early naïve B-cell repopulation as a relapse biomarker in a second cohort and evaluate other predictors using multivariable analysis (MVA) in this larger cohort with a view to developing a proposal for a more effective personalized retreatment algorithm in AAV treated with rituximab induction.

## Methods and Materials

### Patients and Design

A retrospective observational cohort study was conducted of the first 1,000 consecutive rituximab-treated patients with any rheumatological diagnosis in a single center between January 2006 and July 2020. Inclusion criteria were adults (≥18 years old) and fulfilling the Chapel Hill Consensus Conference definitions of systemic vasculitides (17). Exclusion criteria were no clinical and/or B-cell data in cycle 1 (C1) rituximab and receiving repeat cycles in C2 based on fixed-retreatment strategy.

Leeds (West) Research Ethics Committee (REC) confirmed that ethical approval was not required because all treatment decisions were made before evaluation of data, in accordance with the National Health Service (NHS) REC guidelines. The use of off-label rituximab prior to its licensing was approved by Leeds Teaching Hospitals NHS Trust Drug and Therapeutic Committee. To compare baseline B-cell data, results were compared with pre-existing disease controls in rheumatoid arthritis (RA) (*N* = 62) (18) and SLE (*N* = 89) (16) as previously published.

### Treatment

All patients received a first cycle of therapy consisting of 100 mg of methylprednisolone and 1,000 mg of MabThera^®^ on days 1 and 14. Further cycles consisted of the same regimen repeated on clinical relapse (defined below). Continuation of a stable dose or reduction of concomitant immunosuppressant, including oral prednisolone was left to clinicians’ discretion, aiming to stop glucocorticoid if remission was achieved at 6 months. Concomitant cyclophosphamide was used in 5/60 (8.3%) patients with severe organ-threatening AAV.

### Clinical Data and Outcomes

Disease activity was assessed at baseline and every 3 months postrituximab using Birmingham Vasculitis Activity Score (BVAS) version 3.0 (19) without knowledge of B-cell results. Complete response (CR) was defined as BVAS = 0 while partial response (PR) was defined as clinically significant improvement of disease activity without fulfilling the criteria for CR. Relapse was defined as new, reappearance, or worsening of persistent disease (i.e., BVAS increasing by ≥1).

### Laboratory Measures

ANCA staining pattern was determined by indirect immuno-fluorescence, its antigen specificity for myeloperoxidase (MPO) or proteinase-3 (PR3) by Bioplex 2200 Immunoassay and immunoglobulin titers were measured by nephelometry at baseline and every 6 months posttherapy at routine NHS laboratory.

Peripheral blood B-cell subsets (naïve, memory, and plasmablast cells) were quantified using HSFC as a part of routine clinical practice in our department at an accredited Leeds Haematological Malignancy Diagnostic Service clinical laboratory as previously described (20) at weeks 0, 6, 26, and 52 and at clinical relapse without knowledge of clinical status other than time since rituximab. Naïve B cells were defined as CD19^+^CD27^−^CD14^−^CD3^-^ mononuclear cells. Memory B cells were defined as CD19^+^CD27^+^CD38^−^CD14^−^CD3^−^ mononuclear cells (excluding cells gated as plasmablasts). Plasmablasts were defined as CD19^+/−^CD27^+^CD38^++^CD14^−^CD3^−^ mononuclear cells. CD45 was used to calculate absolute cell count. Complete B-cell depletion was defined as a sum of all three subsets below the limit of detection (<0.0001 × 10^9^ cells/L for a white cell count of 5.0 × 10^9^/L) and repopulation as counts above this level.

To compare these HSFC data with a conventional CD19 flow cytometry protocol, CD19^+^ cell count was calculated as the sum of naïve and memory B cells. Detectable CD19 was defined as counts ≥16 cells/µl, a limit of detection typically reported in conventional flow cytometry studies (21).

### Statistical Analyses

At rituximab baseline, peripheral B-cell subsets were compared between patients with AAV, RA, and SLE using Kruskal-Wallis for multiple comparison followed by Mann-Whitney *U* test. For the prediction of clinical relapse in cycle 1 rituximab, multiple imputation by chained equations was used to estimate missing data, and twenty multiple imputation sets were used to provide stability of results. In MVA, only variables with *p* < 0.20 in UVA and two other variables of interest (i.e., concomitant immunosuppressant and BVAS score at baseline) were analysed. The proportional hazard assumption was tested by examining the Kaplan-Meier curves and the Schoenfeld residuals plots. Cox proportional hazards regression was performed using backward elimination, with *p* < 0.20 associated with the deviance used for exclusion from the model. Survival analyses for the categorically distributed biomarkers were calculated using Kaplan-Meier plot and log-rank test.

Receiver operator characteristic (ROC) curves were used to compare the predictive strength of time to relapse using biomarkers between naïve B-cell repopulation and the protocol used in MAINRITSAN2, new or reappearance of ANCA as measured using indirect immunofluorescence or increased titer by at least doubling of either anti-PR3 or anti-MPO antibody and/or CD19^+^ cells return (14) at 6 and 12 months post-therapy. All statistical analysis was performed using Stata MP version 16 and Graph Pad Prism version 8 for Windows.

## Results

### Patient and Treatment Characteristics

**Figure 1:**
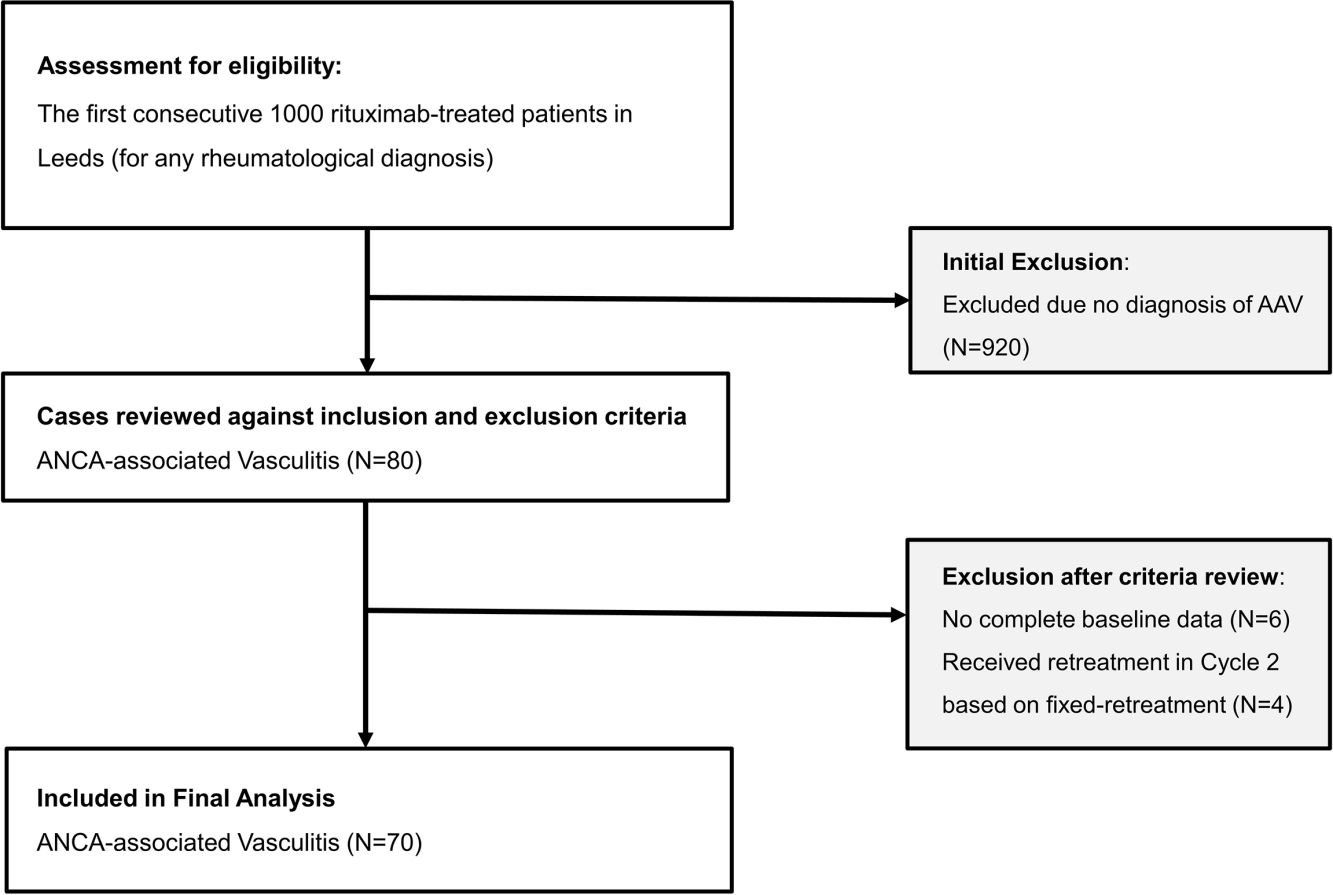
Flow chart of participant into the study.

The flow chart of participant is illustrated in **Figure 1**. A total of 80/1,000 patients had a diagnosis of AAV. Of these, 70 were included in the analysis (published discovery cohort = 35; validation cohort = 35). Four patients were excluded as they were retreated using 6 monthly retreatment following remisison induction due to organ-threatening manifestations while another 6 had no complete baseline data since their care was transferred to our unit later on during rituximab therapy.

Baseline characteristics of the 70 patients with AAV are described in **Table 1**. There was no difference in salient baseline clinical characteristics and laboratory measures apart from slight predominant Caucasians in the validation cohort compared with the published discovery cohort (94.3% vs. 80%, respectively).

**Table 1:** Characteristics/measures of 70 AAV patients at first rituximab infusion.

Characteristics or measures	Discovery cohort (*N* = 35)	Validation cohort (*N* = 35)	Total cohort (*N* = 70)
Age [mean (SD) years]	51 (16.9)	53 (20.2)	52 (18.5)
Male [*N* (%)]	19 (54.3)	19 (54.3)	38 (54.3)
Ethnicity [*N* (%)]			
Caucasian	28 (80.0)	33 (94.3)	61 (87.1)
South Asian	5 (14.2)	2 (5.7)	7 (10.1)
Chinese/South East Asian	1 (2.9)	0	1 (1.4)
Mixed race	1 (2.9)	0	1 (1.4)
Disease duration [median (IQR) years]	2.2 (0.9–5.3)	1.9 (0.4–3.5)	2 (0.6–4.4)
Disease type [*N* (%)]			
Granulomatosis with polyangiitis (GPA)	29 (82.9)	22 (62.9)	51 (72.9)
Microscopic polyangiitis (MPA)	6 (17.1)	10 (28.6)	16 (22.9)
Eosinophilic granulomatosis with polyangiitis (EGPA)	0	3 (8.6)	3 (4.3)
Positive ANCA at diagnosis [No. (%)]	34 (97.1)	30 (85.7)	64 (91.4)
Anti-PR3 antibody	25 (71.4)	19 (54.3)	44 (62.9)
Anti-MPO antibody	5 (14.3)	10 (28.6)	15 (21.4)
Immunofluoresence only	4 (11.4)	1 (2.9)	5 (7.1)
Negative but with a positive histology of GPA/EGPA	1 (2.9)	5 (14.3)	6 (8.6)
Positive anti-PR3/anti-MPO at cycle 1 rituximab infusion [*N* (%)]	26 (74.3)	25 (71.4)	51 (72.9)
Prior/concomitant therapy with cyclophosphamide [*N* (%)]	32 (91.4)	30 (85.7)	62 (88.6)
No. of prior immunosuppressant failure (including Cyclophosphamide and plasma exchange but excluding steroid) [median (range)]	2 (0–5)	2 (0–4)	2 (0–5)
Concomitant immunosuppressant/started within 3 months of cycle 1 rituximab infusion [*N* (%)]	23 (65.7)	23 (65.7)	46 (65.7)
Methotrexate	6 (17.1)	4 (11.4)	10 (14.3)
Azathioprine	8 (22.9)	11 (31.4)	19 (27.1)
Mycophenolate mofetil	9 (25.7)	6 (17.1)	15 (21.4)
Cyclophosphamide^a^	2 (5.7)	3 (8.6)	5 (7.1)
Tacrolimus	0	1 (2.9)	1 (1.4)
Concomitant oral prednisolone [*N* (%)]	30 (85.7)	32 (91.4)	62 (88.6)
Oral prednisolone dose [mean (SD), mg/day]	13 (9.6)	23 (13.3)	18 (12.6)
Organ system involvement [*N* (%)]			
Ear, nose, and throat (ENT)	25 (71.4)	23 (65.7)	48 (68.6)
Musculoskeletal and general	20 (57.1)	22 (62.9)	21 (58.3)
Chest	16 (45.7)	17 (48.6)	33 (47.1)
Renal	12 (34.3)	13 (37.1)	25 (35.7)
Mucocutaneous	8 (22.9)	6 (17.1)	14 (20)
Nervous system	3 (8.6)	6 (17.1)	9 (12.9)
Eyes	6 (17.1)	3 (8.6)	9 (12.9)
Abdominal	1 (2.9)	1 (2.9)	2 (2.9)
BVAS 3.0 score [mean (SD)]	10.5 (5.9)	11.5 (5.5)	11 (5.7)
VDI score (median (range)]	0 (0–5)	1 (0–5)	1 (0–5)
Immunoglobulin level [mean (SD), g/dl]			
IgM (normal range, 0.5–2.0 g/L)	0.95 (0.67)	0.91 (0.85)	0.93 (0.76)
IgA (normal range, 0.8–4.0 g/L)	2.22 (1.35)	1.73 (0.79)	1.97 (1.13)
IgG (normal range, 6.0–16.0 g/L)	10.03 (4.92)	8.86 (3.86)	9.44 (4.43)
Lymphocyte count [mean (SD), ×10^9^/L] (normal range 1.00–4.50)	1.35 (0.65)	1.10 (0.63)	1.2 (0.6)
Total B cells [median (IQR), ×10^9^ cells/L]	0.0402 (0.0181–0.0835)	0.0512 (0.0144–0.1741)	0.0410 (0.0160–0.1200)
Naïve B cells [median (IQR), ×10^9^ cells/L]	0.0259 (0.0086–0.0540)	0.0275 (0.0060–0.1021)	0.0259 (0.0075–0.0782)
Memory B cells [median (IQR), ×10^9^ cells/L]	0.0148 (0.0057–0.0331)	0.0129 (0.0045–0.0358)	0.0132 (0.0055–0.0344)
Plasmablasts [median (IQR), ×10^9^ cells/L]	0.0021 (0.0011–0.0032)	0.0014 (0–0.0033)	0.0018 (0.0007–0.0032)
CRP [mean (SD), mg/L]	29.1 (37.4)	27.1 (37.5)	28.1 (37.2)
Total B-cell counts [median (interquartile range), ×10^9^ cells/L]			
Group 1: Patients without concomitant oral immunosuppressant	0.0519 (0.0713)	0.0584 (0.2244)	0.0551 (0.1115)
Group 2: Patients with concomitant oral immunosuppressant	0.0370 (0.0641)	0.0362 (0.1582)	0.0369 (0.0789)
Difference between groups	*p* = 0.899	*p* = 0.232	*p* = 0.509
Total B-cell counts [median (interquartile range), ×10^9^ cells/L]			
Group 1: Patients without concomitant oral prednisolone	0.0445 (0.0399)	0.1708 (0.1923)	0.0583 (0.1338)
Group 2: Patients with concomitant oral prednisolone	0.0402 (0.0804)	0.0362 (0.1511)	0.0399 (0.1070)
Difference between groups	*p* = 0.659	*p* = 0.226	*p* = 0.171

BVAS, Birmingham Vasculitis Activity Score version 3.0; IS, immunosuppressant; rituximab, rituximab; VDI, Vasculitis Damage Index.

a Combination of rituximab and 2–4 pulses of intravenous cyclophosphamide were administered for remission induction of severe AAV to 5 patients with critical subglottic stenosis (N = 3), renal involvement with rapidly rising serum creatinine (N = 1), and probable cardiac involvement (N = 1).

A total of 282 rituximab cycles were administered during a total follow-up of 535.3 patient-years (PYs). Median (IQR) duration of follow-up per patient was 7.1 years (4.5–11.1).

### Clinical Response

A high rate of clinical response (PR or CR) at 6 months were observed; rates for cycles C1–5 were 68/70 (97.1%), 55/57 (96.5%), 36/41 (87.8%), 24/27 (88.9%), and 18/20 (90%), respectively.

The duration of response in rituximab responders was considerably longer than 26 weeks; median (range) time-to-rituximab retreatment for C1–5 were 84 weeks (39–402), 73 weeks (39–246), 67 weeks (38–156), 60 weeks (40–196), and 73 weeks (42–263), respectively, thus indicating that a 6-month interval for fixed-schedule dosing is unnecessarily short for the majority of patients.

Details about long-term efficacy and safety of retreatment on clinical relapse strategy are described in the **Online Supplementary File**, **Figure S1** and **Table S2**.

### Relapse

In C1, 59/70 (84.3%) patients had experienced a clinical relapse. We next analyzed relapse episodes in the first five rituximab cycles since these would roughly equate to the number of courses given in the fixed-schedule dosing group in the MAINRITSAN protocol.

In C1–5 with a follow-up of 467 PYs, there were 158 relapse episodes in 60 patients. Of these, 16 were major relapses in 15 patients (renal = 7; neurology = 4; ears, nose, and throat (ENT) = 3; respiratory = 2) (**Online Supplementary Table S1**). The rate of major relapse was 3.4/100 PY. The majority of major relapses were retreated with rituximab and glucocorticoids apart from two patients who were treated with intravenous cyclophosphamide and one with plasma exchange.

### Comparison of B-Cell Signatures Across Three Diseases at Rituximab Initiation

**Figure 2:**
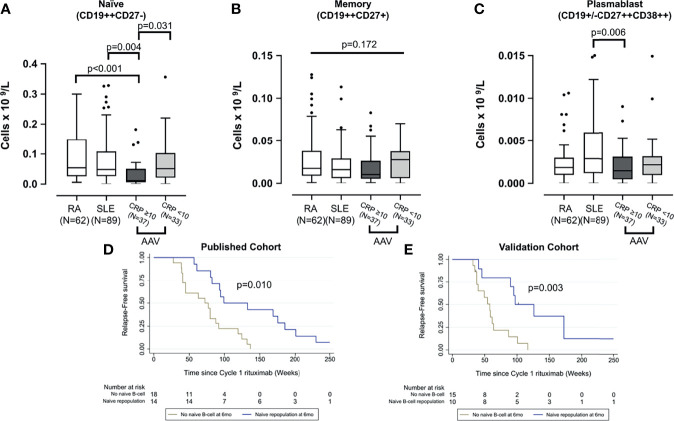
Comparison of peripheral B-cell subsets across three diseases and validation of B-cell biomarkers of relape. B-cell subsets including naïve **(A)**, memory **(B)**, and plasmablast **(C)** were compared between patients with rheumatoid arthritis, systemic lupus erythematosus, and AAV at rituximab initiation. The latter was divided into those with and without severe systemic inflammation; raised CRP (i.e., >10 mg/L). The box plots denote median, and the error bars represent Tukeys. Analyses were performed using Kruskal-Wallis followed by Mann-Whitney *U* test. Naïve B-cell repopulation at 6 months as a biomarker of later relapse was analyzed using Kaplan-Meier survival analysis in both the published discovery cohort **(D)** and the validation cohort **(E)**.

Prior to first rituximab infusion, naïve and plasmablast cells differed between AAV, RA, and SLE groups (*p* < 0.001 and *p* = 0.018, respectively) using Kruskal-Wallis test. The data from this larger AAV cohort reconfirmed our previous finding, that active AAV is associated with naïve lymphopenia, and this effect is stronger if CRP was raised (i.e., ≥10 mg/L; *p* = 0.031). Naïve B cells were also lower in active AAV compared with RA and SLE **(** **Figure 2A** **)**. While there was no difference in memory B cell between the three diseases (*p* = 0.172) **(** **Figure 2B** **)**, plasmablasts were higher in SLE than active AAV (*p* = 0.006) **(** **Figure 2C** **)**.

### Validation of Naïve B-Cell Repopulation as a Biomarker of Longer Time to Relapse

The published discovery cohort included 32/35 AAV patients with complete B-cell data (3). In this validation cohort, 25/35 subsequent and consecutive patients with B-cell data available were analyzed. Similar to the discovery cohort **(** **Figure 2D** **)**, the Kaplan-Meier survival analysis showed a significant association between repopulation of naïve B cells at 6 months and longer time to relapse (*p* = 0.003) in this validation cohort **(** **Figure 2E** **)**.

### Predictors of Time to Relapse to First-Cycle Rituximab

Baseline and 6-month variables were analyzed as predictors of relapse in patients who responded to rituximab. Complete B-cell data were available in 57/70 patients. In imputed MVA, concomitant immunosuppressant HR [0.48 (95% CI, 0.24–0.94)], achieving CR at 6 months [0.24 (0.12–0.50)], and naïve repopulation at 6 months [0.43 (0.22–0.84)] were associated with longer time to relapse. Higher baseline memory B cells were associated with shorter time to relapse [1.01 (1.00–1.02)] **(** **Table 2** **)**.

**Table 2:** Factors associated with time to relapse to first cycle rituximab.

Risk factors	Univariable analysis	Multivariable analysis (MVA)
	HR (95% CI); *p*-values (with multiple imputation)	HR (95% CI); *p*-values (with multiple imputation)
**Baseline clinical/serological characteristics**		
Age at rituximab initiation (per 10 years)	1.01 (0.86–1.17); *p* = 0.954	Not included in MVA
Female	1.15 (0.65–2.02); *p* = 0.629	Not included in MVA
Disease duration at rituximab initiation (years)	1.06 (0.98–1.15); *p* = 0.160	Included in MVA but removed from final model as *p* < 0.20
Concomitant immunosuppressant	0.69 (0.39–1.22); *p* = 0.205	**0.48 (0.24–0.94); *p* = 0.034**
Positive ANCA immunofluorescence	0.89 (0.46–1.71); *p* = 0.725	Not included in MVA
Positive anti-PR3/anti-MPO at rituximab initiation	0.57 (0.31–1.06); *p* = 0.077	Included in MVA but removed from final model as *p* < 0.20
CRP at ri initiation (mg/L)	1.00 (0.99–1.01); *p* = 0.456	Not included in MVA
BVAS 3.0 per point score	0.99 (0.94–1.05); *p* = 0.763	Included in MVA but removed from final model as *p* < 0.20
VDI per point score	1.14 (0.87–1.50); *p* = 0.353	Not included in MVA
**Clinical and serological characteristics at 26 weeks**		
Complete response	**0.34 (0.19**–**0.61); p<0.001**	**0.24 (0.12**–**0.50); p<0.001**
Positive ANCA immunofluorescence	0.99 (0.56–1.75); *p* = 0.962	Not included in MVA
Positive anti-PR3/anti-MPO	0.79 (0.44–1.42); *p* = 0.426	Not included in MVA
CRP (mg/L)	0.99 (0.97–1.02); *p* = 0.618	Not included in MVA
**B-cell subsets, depletion, and repopulation**		
Naïve B cells at rituximab initiation (×10^9^/L)^a^	1.00 (1.00–1.01); *p* = 0.797	Not included in MVA
Memory B cells at rituximab initiation (×10^9^/L)^a^	**1.01 (1.00–1.02); *p* = 0.040**	**1.01 (1.00–1.02); *p* = 0.045**
Plasmablasts at rituximab initiation (×10^9^/L)^a^	1.04 (0.94–1.16); *p* = 0.459	Not included in MVA
Complete depletion at 6 weeks postrituximab	0.90 (0.50–1.61); *p* = 0.721	Not included in MVA
**Naïve B-cell repopulation at 26 weeks**	**0.38 (0.19–0.76); *p* = 0.006**	**0.43 (0.22–0.84); *p* = 0.013**
Memory B-cell repopulation at 26 weeks	**0.45 (0.20–0.99); *p* = 0.046**	Included in MVA but removed from final model as *p* < 0.20
Plasmablast cell repopulation at 26 weeks	1.14 (0.61–2.13); *p* = 0.675	Not included in MVA

a (Count ×10^9^ cells/L) for each subset multiplied by 1,000 prior to analysis. The bold values denote variables which are statistically significant in the analyses.

### Comparison of Relapse Prediction Based on Naïve Repopulation Versus ANCA and/or CD19+ Cell Return at 6 Months

In order to estimate the likelihood effectiveness of different personalized treatment strategies, we compared the accuracy of relapse prediction based on ANCA and/or total CD19^+^ cell return (according to a conventional flow cytometry protocol) as per MAINRITSAN2 versus prediction based on absent naïve B cells using HSFC. At 6 months postrituximab, the proportion of patients with anti-PR3/anti-MPO positivity had reduced from 50/70 (71.4%) to 24/70 (34.3%) (*p* < 0.001). No patient had new or worsening of ANCA titers. Only 3/57 (5.3%) patients had detectable CD19^+^ cells based on conventional flow cytometry whereas CD19^+^ cells were detected in 31/57 (54.4%) if enumerated using HSFC.

**Figure 3:**
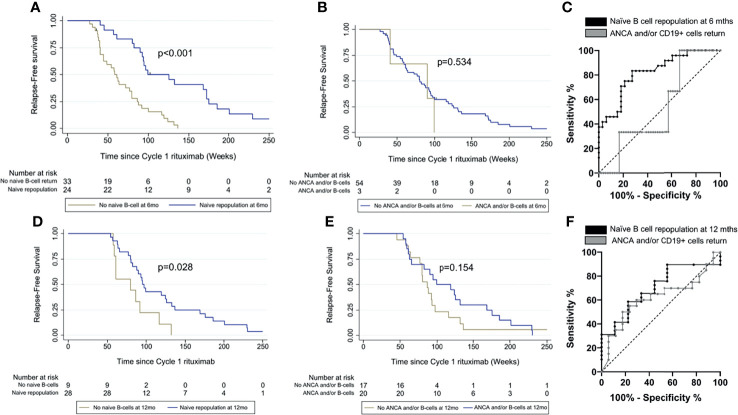
Comparison of relapse prediction based on naïve B cells and ANCA and/or CD19+ cell return. Time to relapse was compared between patients with and without naïve repopulation in **(A)** at 6 months and **(D)** at 12 months and between patients with and without ANCA and/or CD19+ cells return in **(B)** at 6 months and **(E)** at 12 months respectively using Kaplan-Meier survival analyses. Areas under the receiver operating characteristic (AUROC) were compared between the two biomarker-led retreatment strategies **(C)** at 6 months and **(F)** at 12 months postrituximab.

Using HSFC, patients with naïve B-cell repopulation at 6 months had longer time to relapse compared with those without naïve repopulation (*p* < 0.001) **(** **Figure 3A** **)**. Relapse rates at 12 and 18 months were 2/24 (8%) and 4/24 (17%) with naïve repopulation at 6 months and 13/33 (39%) and 20/33 (61%) without naïve repopulation. In contrast, there was no difference in time to relapse between those with or without ANCA and/or CD19^+^ return at 6 months (*p* = 0.534), although the analysis was limited by only 3/48 patients in the former **(** **Figure 3B** **)**.

The area under the ROC (AUROC) curve for time to relapse was greater for naïve B-cell repopulation using HSFC compared with absence of ANCA and/or CD19^+^ return using conventional flow cytometry at 6 months, 0.82 (95% CI, 0.71–0.93) and 0.53 (0.26–0.80), respectively **(** **Figure 3C** **)**.

### Comparison of Relapse Prediction Based on Naïve Repopulation Versus ANCA and/or CD19^+^ Cells Return at 12 Months

Of 59/70 patients who had a clinical relapse in cycle 1 rituximab, 44/59 had not yet relapsed at 12 months. Data for B cells and ANCA were available in 37/44 patients for analysis.

At 12 months postrituximab, only 11/37 (30%) patients had either reappearance of ANCA or increased titer by at least doubling of either anti-PR3 or anti-MPO antibody. A total of 12/37 (32%) patients had CD19^+^ cells detectable as defined by a conventional cytometry protocol. The total number of patients with ANCA and/or CD19^+^ cell return was 20/37. Using HSFC, 28/37 patients had naïve B-cell repopulation and 9/37 lacked naïve repopulation at 12 months. Of 11/37 patients with reappearance of ANCA or increased antibody titer, 9/11 had naïve repopulation at 12 months.

Using HSFC, patients with naïve B-cell repopulation at 12 months had longer time to relapse compared with those without naïve repopulation (*p* = 0.028) **(** **Figure 3D**). Relapse rates at 18 and 24 months were 6/28 (21%) and 16/28 (57%) with naïve repopulation and 4/9 (44%) and 7/9 (78%) without repopulation at 12 months. There was no association between ANCA and/or CD19^+^ cells return and longer time to relapse (*p* = 0.154) **(** **Figure 3E** **)**.

The AUROC for time to relapse was greater for naïve B-cell repopulation using HSFC compared with absence of ANCA and/or CD19^+^ return using conventional flow protocol at 12 months, 0.70 (95% CI, 0.52–0.88) and 0.62 (0.43–0.80), respectively **(** **Figure 3F** **).**

### Proposed Algorithm for Personalized Rituximab Retreatment Based on Clinical and B-Cell Biomarkers

**Figure 4:**
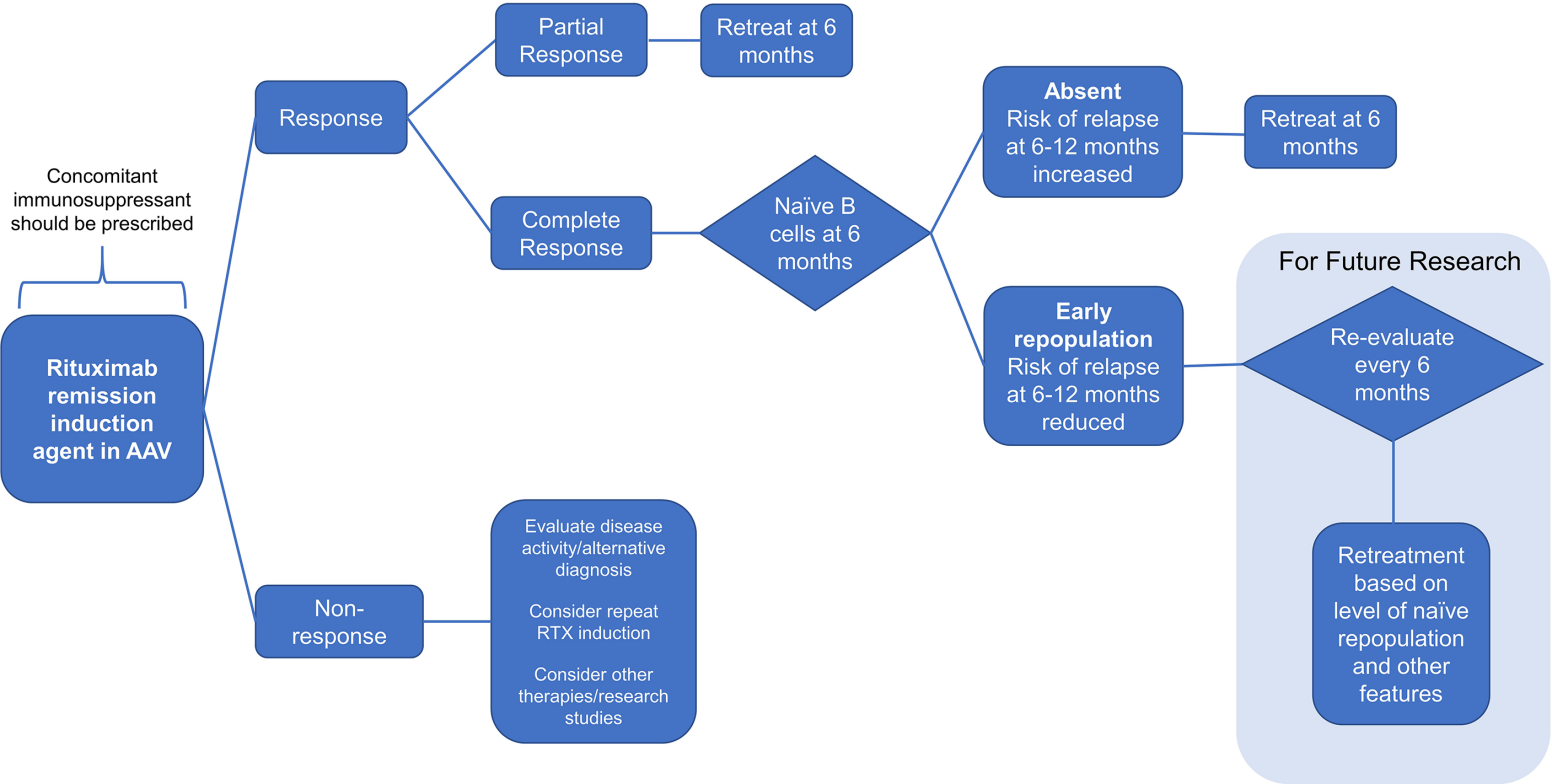
Flow diagram. A proposal for personalized rituximab retreatment algorithm based on clinical predictors and early naïve B-cell return in ANCA-associated vasculitis.

Based on the results above, we propose an algorithm for personalized rituximab retreatment as illustrated in **Figure 4**. Our data suggest that the key decisions for sustained response at 6 months are as follows: (i) use of concomitant immunosuppressants; (ii) retreatment if clinical response is incomplete; and (iii) retreatment if naïve B-cell repopulation is not detected. In our cohort, this would have led to retreatment at 6 months in 47/62 (76%) of patients, with the remainder not requiring fixed retreatment. Further research is needed to characterize the use of clinical and B-cell biomarkers beyond the 6-month time-point as our sample size is currently insufficient to address this question.

## Discussion

In this study, we further characterized the use of naïve B cell (as enumerated by HSFC) as a disease-specific biomarker to guide rituximab retreatment decisions in AAV alongside new data on clinical predictors of relapse.

Our key finding, that B-cell return was associated with sustained response, may initially appear counterintuitive. However, repopulation of nonautoimmune B cells is desirable and would lead to the observed repopulation with naïve cells (22). Naïve B cells are produced by the bone marrow constantly and will become detectable as soon as the serum concentration of rituximab becomes too low to kill them. Repopulation of naïve B cells is an expected and healthy outcome of rituximab therapy and does not indicate recrudescence of autoimmunity (23, 24). Other factors in B-cell homeostasis and function may also be considered in understanding our results. We only monitor B cells in peripheral blood, but in fact these cells traffic between bone marrow, inflamed tissues, and secondary lymphoid tissues. The numbers measured in blood may not correlate with these other sites, which are perhaps more clinically relevant (25). Next, some investigators have proposed that IL-10-producing regulatory B cells may be important in autoimmunity. CD19^+^CD24^hi^CD38^hi^27^−^ cells are regulatory B cells which have previously been identified within the transitional B-cell subsets. This is part of the naïve B-cell gate that we associate with maintenance of remission in AAV (26).

The predictive value of naïve B-cell repopulation in relapse prediction may not only be useful at 6 months but also predict outcomes at 12 months postrituximab if retreatment was not already given. Despite having only a small number of patients without naïve repopulation at 12 months available for comparison (i.e., the majority of those without naïve repopulation at 6 months had been retreated within 12 months of rituximab), our results still showed that repopulation and higher naïve B-cell numbers were associated with longer time to relapse. This finding requires further work to confirm, which will be done in future analyses of our cohort. Although higher baseline memory B cells were predictive of shorter time to relapse in MVA, its effect size was the smallest compared with the other three significant predictors in the model.

A few studies have reported predictors of relapse to rituximab, but these data were analyzed using cohort treated with mixed retreatment strategies (27, 28). Using retreatment-on-relapse strategy, our cohort is unique and valuable for discovery of novel biomarkers and other clinical predictors of relapse. First, patients who were coprescribed immunosuppressant had longer time to relapse. A previous randomized controlled trial supported the continuation of immunosuppressant for long-term remission maintenance and improved renal survival in AAV (29). Consistent with this, concomitant immunosuppressant has been shown to prolong duration of response in randomized studies in other B-cell-mediated diseases like RA (30, 31). Second, patients with incomplete response had earlier relapse, suggesting they should have early retreatment, both to prevent relapse and to improve their level of response. The latter point is consistent with data from RA, in which patients with incomplete or nonresponse to a first cycle of rituximab had improved response after retreatment at 6 months (18, 32). Third, in the current study, no added value of ANCA monitoring up to 12 months was found, since no patient had changes in ANCA at 6 months while the majority of patients with ANCA changes at 12 months (i.e., 9/11) also had naïve repopulation. Our data are therefore consistent with previous reports on the limited value of ANCA in guiding retreatment (33, 34).

Herein, we therefore propose a personalized rituximab retreatment regimen; that all patients should be coprescribed an oral immunosuppressant with rituximab therapy; patients with PR at 6 months should be retreated pre-emptively with rituximab at 6 months; and patients with CR at 6 months and no repopulation of naïve B cell at 6 months receive retreatment at 6 months. Patients with CR and naïve B-cell return at 6 months should not receive fixed retreatment and should be monitored for a further 6 months. Applying this algorithm to our own cohort would have avoided an unnecessary fixed retreatment in 24% of patients without allowing those patients to relapse in the subsequent 6 months.

This study has some limitations. First, B-cell data were missing for a small number of patients due to their nonattendance for review at the 6-month time-point. As these were deemed missing at random, multiple imputation was used to reduce potential bias in parameter estimation as well as enhancing generalizability of the results. Second, concomitant immunosuppressant was used in about two-thirds of the patients, thus efficacy could not be attributed to rituximab alone. Concomitant immunosuppressants showed an association with time to relapse but were not prescribed in a randomized fashion. Importantly, there was no difference in either lymphocyte or B-cell numbers between those with and without concomitant immunosuppressant at rituximab baseline. Third, 73% of our patients had granulomatosis with polyangiitis (GPA), hence our proposed algorithm may not be generalized to those with microscopic polyangiitis or eosinophilic GPA predominant. Fourth, the remission induction agent used in this study was rituximab. Our results therefore cannot be generalized to patients who received cyclophosphamide induction followed by rituximab maintenance. Lastly, in terms of clinical applicability, we acknowledge that B cells are not routinely measured in every department. If only complete remission at 6 months was used for predicting relapse, this algorithm would avoid retreatment at 6 months in 38/70 (54%) patients but with 7/38 (18%) relapse rate at 12 months postrituximab. Therefore, our results showed the added value of naïve B-cell monitoring in reducing frequency of retreatment without allowing those patients to relapse within 12 months of rituximab therapy. Future health economic studies will ascertain the cost-effectiveness of B-cell monitoring in AAV patients treated with rituximab.

In conclusion, this observational study has led to a proposal for a rituximab retreatment algorithm that should be evaluated in interventional trials.

## Data Availability Statement

The original contributions presented in the study are included in the article/**Supplementary Material**. Further inquiries can be directed to the corresponding author.

## Ethics Statement

Ethical review and approval was not required for the study on human participants in accordance with the local legislation and institutional requirements. The patients/participants provided their written informed consent to participate in this study.

## Author Contributions

JA, EV, PE, and MYMY: substantial contributions to the conception and design of the work, the acquisition, analysis, and interpretation of data, drafting the work or revising it critically for important intellectual content, and final approval of the version published. SD, AA, AR, and SS: substantial contributions to the acquisition and interpretation of data, drafting the work or revising it critically for important intellectual content, and final approval of the version published. All authors have agreed to be accountable for all aspects of the work in ensuring that questions related to the accuracy or integrity of any part of the work are appropriately investigated and resolved.

## Funding

This research was funded/supported by the the Wellcome Trust Institutional Strategic Support Fund to MYMY (204825/Z/16/Z), National Institute for Health Research (NIHR) Doctoral Research Fellowship to MYMY (DRF-2014-07-155), and NIHR Clinician Scientist to EV (CS-2013-13-032). This article/paper/report also presents independent research funded/supported by the NIHR Leeds Biomedical Research Centre. The views expressed are those of the author(s) and not necessarily those of the NIHR or the Department of Health and Social Care.

## Conflict of Interest

SD has received honoraria from Roche and GSK. SS has received honoraria from Novartis, Swedish Orphan Biovitrum (SOBI), and Sire and grant support from Novartis, Swedish Orphan Biovitrum, Octapharma, and CSL Behring. EV has received honoraria and research grant support from Roche, GSK, and AstraZeneca. PE has received consultant fees from BMS, Abbott, Pfizer, MSD, Novartis, Roche, and UCB. He has received research grants paid to his employer from Abbott, BMS, Pfizer, MSD, and Roche. MYMY has received consultancy fees from Aurinia Pharmaceuticals.

The remaining authors declare that the research was conducted in the absence of any commercial or financial relationships that could be construed as a potential conflict of interest.

## Publisher’s Note

All claims expressed in this article are solely those of the authors and do not necessarily represent those of their affiliated organizations, or those of the publisher, the editors and the reviewers. Any product that may be evaluated in this article, or claim that may be made by its manufacturer, is not guaranteed or endorsed by the publisher.
