# Supplementary material for: A Personalized Rituximab Retreatment Approach Based on Clinical and B-Cell Biomarkers in ANCA-Associated Vasculitis
Source: Front Immunol. 2022 Jan 12;12:803175. doi: 10.3389/fimmu.2021.803175 (PMC8789753; doi:10.3389/fimmu.2021.803175)
Supplement: Supplementary file 1 [file DataSheet_1.docx]

**SUPPLEMENTARY DATA**

**A proposal for individually tailored rituximab retreatment based on clinical and B-cell biomarkers for remission maintenance in ANCA-associated vasculitis**

**CONTENTS:**

1. Table S1: Main characteristics of the 16 major relapses using tailored rituximab based on clinical relapse in AAV
2. Details on long-term efficacy and safety of retreatment on clinical relapse strategy
3. Figure S1: Long-term efficacy and safety of retreatment on clinical relapse strategy
4. Table S2: IgG levels measured at 6 months of each cycle of rituximab infusion

**Table S1: Main characteristics of the 16 major relapses using tailored rituximab based on clinical relapse in AAV**

| **Diagnosis, Flare Type** | **Age, Gender** | **ANCA status** | | **Concomitant IS at Relapse** | **Relapse Characteristics and Treatment** | **RTX Cycle with Major relapse** | **Time of Relapse since last RTX** |
| --- | --- | --- | --- | --- | --- | --- | --- |
|  |  | **Cycle 1 RTX** | **Major Relapse** |  |  |  |  |
| MPA, relapsing disease | 55, F | MPO +ve | MPO +ve | Azathioprine | **Major:** Renal (Haematuria)  **Minor:** myalgia, arthritis, bloody nasal discharge, conductive deafness, wheeze, hypertension, proteinuria  **Relapse Treatment:** RTX | 2 | 77 weeks |
| GPA, relapsing disease | 27, M | PR3 +ve | PR3 +ve | Methotrexate | **Major:** Renal (Haematuria)  **Minor:** Proteinuria  **Relapse Treatment:** RTX | 2 | 175 weeks |
| GPA, relapsing disease | 62, M | PR3 +ve | PR3 +ve | Mycophenolate Mofetil | **Major:** Renal (Haematuria)  **Minor:** Proteinuria  **Relapse Treatment:** RTX | 2 | 72 weeks |
| GPA, relapsing disease | 59, F | PR3 +ve | PR3 +ve | Azathioprine | **Major:** Renal (Haematuria)  **Minor:** Creatinine 250-499 μ/L  **Relapse Treatment:** RTX | 2 | 229 weeks |
| MPA, relapsing disease | 57, F | MPO +ve | Negative | Mycophenolate Mofetil | **Major:** ENT (sensorineural hearing loss)  **Minor:** Worsening peripheral neuropathy  **Relapse Treatment:** RTX | 2 | 132 weeks |
| MPA, relapsing disease | 39, M | MPO +ve | MPO +ve | Azathioprine | **Major:** Renal (Haematuria, Rise in serum creatinine >30% or fall in creatinine clearance >25%)  **Minor:** Proteinuria  **Relapse Treatment:** RTX. Progression to end-stage renal failure requiring renal replacement therapy. | 2 | 34 weeks |
| GPA, Newly diagnosed | 79., F | PR3 +ve | Negative | None | **Major:** ENT (sensorineural hearing loss)  **Minor:** Sinusitis, epistaxis, polyneuropathy, weight loss  **Relapse Treatment:** RTX | 2 | 129 weeks |
| GPA, relapsing disease | 52, M | PR3 +ve | Negative | Mycophenolate Mofetil | **Major:** ENT (sensorineural hearing loss)  **Minor:** Sinusitis  **Relapse Treatment:** RTX | 3 | 245 weeks |
| MPA, relapsing disease | 20, F | Negative | Negative | Mycophenolate Mofetil | **Major:** Neurology (mononeuritis multiplex)  **Minor:** Arthralgia, wheeze, sinusitis  **Relapse Treatment:** RTX | 3 | 46 weeks |
| GPA, relapsing disease | 44, M | MPO +ve | MPO +ve | Mycophenolate Mofetil | **Major:** Renal (Haematuria)  **Minor:** Proteinuria  **Relapse Treatment:** RTX | 3 | 56 weeks |
| GPA, relapsing disease | 74, M | PR3 +ve | PR3 +ve | Mycophenolate Mofetil | **Major:** Neurology (cranial nerve lesion on MRI)  **Relapse Treatment:** RTX | 3 | 157 weeks |
| GPA, relapsing disease | 68, M | Negative | Negative | Methotrexate | **Major:** Respiratory **(**major pulmonary haemorrhage requiring endobronchial stent)  **Minor:** Sinusitis, headache  **Relapse Treatment:** Switched to IV Cyclophosphamide | 3 | 84 weeks |
| GPA, relapsing disease | 49, F | PR3 +ve | PR3 +ve | Azathioprine | **Major:** Respiratory Failure  **Minor:** New lung cavities and nodules, sinusitis  **Relapse Treatment:** Switched to plasma exchange but unfortunately died in hospital with respiratory failure. | 3 | 230 weeks |
| GPA, relapsing disease | 74, M | PR3 +ve | PR3 +ve | Methotrexate | **Major:** Renal (haematuria)  **Minor:** Fever, weight loss, proteinuria  **Relapse Treatment:** RTX | 4 | 149 weeks |
| GPA, relapsing disease | 65, F | Negative | Negative | IVIG | **Major:** Cranial nerve palsy lesion on MRI  **Minor:** Sinusitis  **Relapse Treatment:** RTX | 5 | 139 weeks |
| GPA relapsing disease | 81, M | PR3 +ve | PR3 +ve | Mycophenolate Mofetil | **Major:** Neurology (mononeuritis multiplex)  **Minor:** Proteinuria  **Relapse Treatment:** Switched to IV cyclophosphamide | 5 | 116 weeks |

**Details on long-term efficacy and safety of retreatment on clinical relapse strategy**

BVAS 3.0 score at each subsequent relapse was less severe than at rituximab (RTX) baseline **(Figure S2A).** Over 10 years follow-up, only 13/70 patients (18.6%) discontinued RTX [switched back to intravenous cyclophosphamide due to major relapse=2; progression to end-stage renal failure requiring renal replacement therapy=2; deaths=9 (infection-associated with low IgG=2; pneumonia=3; major cardiovascular event=1; heart failure=1; renal failure=1; respiratory failure=1)]. The 10-year RTX retention survival is illustrated in **Figure S2B.**

Mean daily oral prednisolone dose requirement was substantially reduced; remaining below baseline levels throughout subsequent cycles **(Figure S2C)**, with 17/62 (27.4%) patients who were on concomitant prednisolone at RTX baseline discontinued therapy at the last follow-up. With regards to the effect of concomitant oral prednisolone on B-cells, there were no differences in total B-cell counts between those who were or were not on concomitant oral prednisolone at rituximab baseline **(Table 1)** and at 6 months post-rituximab; p=0.546 (Mann-Whitney Test).

The proportion of patients with an increase in VDI at 6 months after cycle 1 and at relapse of each cycle was as follows: C1: 10/70 (14%); C2: 4/59 (7%); C3: 4/41 (10%); C4: 3/30 (10%); C5: 1/19 (5%). In the majority of the cases, VDI score increased by 1 point. No patient progressed to severe organ damage (i.e. VDI score of ≥5). Two patients who had VDI=5 at baseline had no further increase at the end of the follow-up. Although, some patients had increased in VDI score over time, this might be expected in a cohort of relapsing disease. The VDI also was not able to distinguish accurately whether the damage items scored were due to vasculitis or side effect from therapies including glucocorticoid.

In terms of long-term safety, there were 33 severe infection episodes (SIEs) in 23 patients in a total follow-up of 535.3 PYs. The rate of SIE was 6.2/100 PY. The type of infections were lower respiratory tract=21; urinary tract infection=3; cellulitis/wound infection=3; biliary sepsis=2; neutropenic sepsis=2; line infection=1; and ENT infection=1. In terms of atypical infection, one patient had *mycobacterium gordonae* and one had *pneumocystis jirovecii pneumonia*. No patient developed a progressive multi-focal leukoencephalopathy.

IgG levels were significantly reduced following initial remission induction with cyclophosphamide therapy but the levels remained stable using RTX retreatment on clinical relapse strategy (**Figure S2D**). At the last follow-up, 28/70 (40%) had low IgG level (<6.0 g/L). However using retreatment on clinical relapse, only 13/70 (18.6%) patients had new low IgG during RTX therapy at the last follow-up (**Table S2**). Notably, only 5/70 (7.1%) patients in our cohort required immunoglobulin replacement therapy. 11/28 (39.3%) patients with low IgG level had SIE. Of those on concomitant immunosuppressant, 14/44 (31.8%) patients had SIE.

Prophylactic treatment with co-trimoxazole was not prescribed routinely during therapy with RTX in our unit. There was one death due to arrhythmia and *pneumocystis jirovecii pneumonia* observed during the study. The infection occurred 3 months post Cycle 3 rituximab.

**
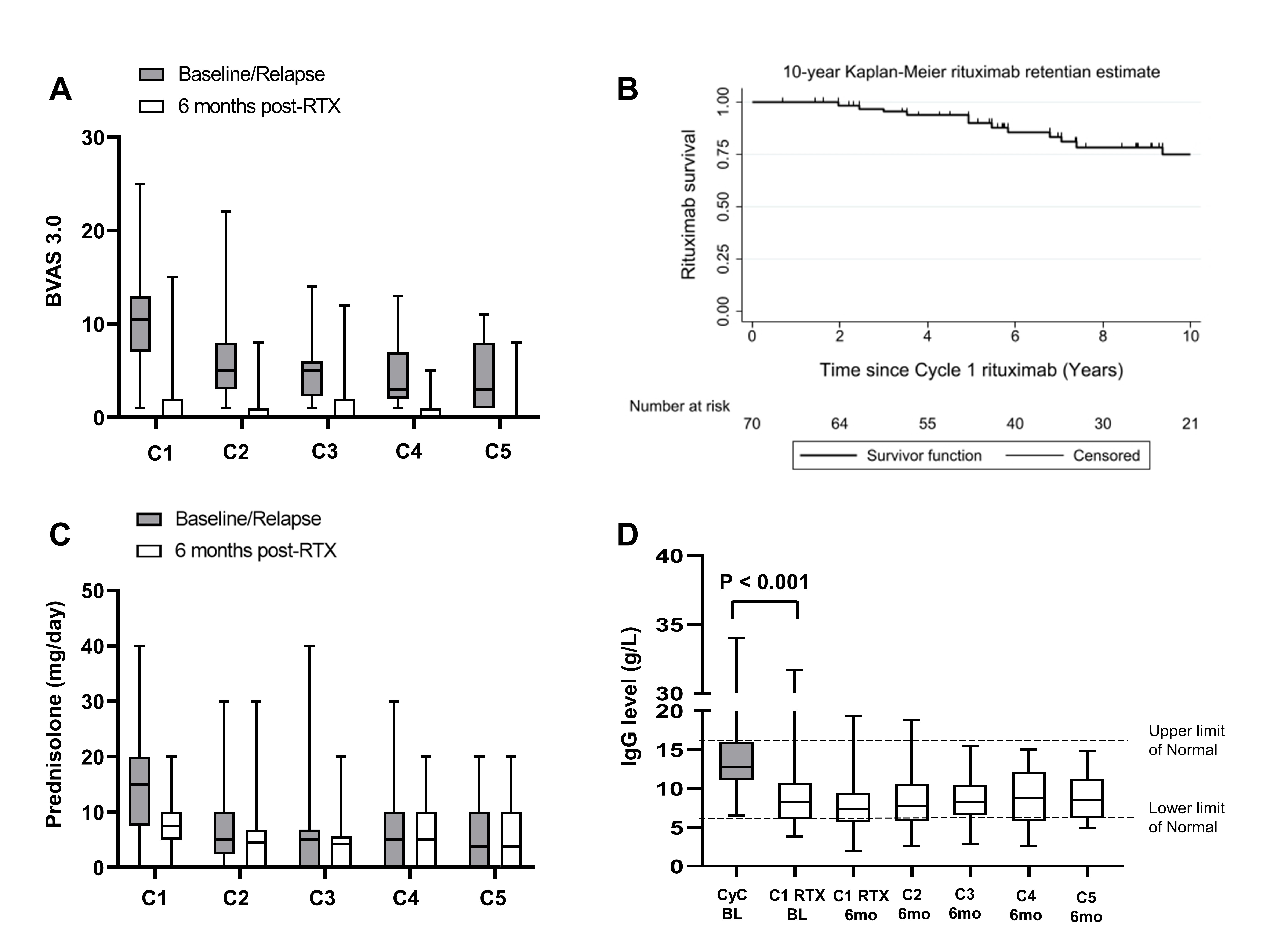
**

**Figure S1: Long-term efficacy and safety of retreatment on clinical relapse strategy.** A) BVAS 3.0 scores at baseline, at relapse and 6-month post-RTX over the first five cycles. B) Kaplan-Meier survival graph of RTX retention over 10-year observation. C) Daily oral prednisolone dose requirement over five cycles. D) IgG levels as measured at first treatment with intravenous cyclophosphamide and follow-on therapy with RTX. The dotted lines represent upper and lower limit of normal values. All box-plot represent mean and errors bars denote minimum and maximum values.

**Table S2: IgG levels measured at 6 months of each cycle of rituximab infusion**

| Time point | No. patients  treated in RTX Cycle | No. patients with IgG data available | Mean IgG  (SD), g/L | p value (versus  previous Cycle)* | No. patients with low IgG in RTX Cycle (%)**  [Normal range: 6-16 g/L] | No. Patients with new low IgG since previous RTX Cycle (%) |
| --- | --- | --- | --- | --- | --- | --- |
| Baseline | 70 | 68 | 9.30 (4.4) | N/A | 14/68 (20.6) | 0 (0) |
| Cycle 1 6 months | 70 | 63 | 8.05 (3.6) | 0.003 | 20/63 (31.7) | 10/63 (15.9) |
| Cycle 2 6 months | 60 | 48 | 8.14 (3.1) | 0.277 | 12/48 (25) | 1/48 (2.1) |
| Cycle 3 6 months | 43 | 33 | 8.44 (3.4) | 0.613 | 5/33 (15.2) | 1/33 (3) |
| Cycle 4 6 months | 30 | 21 | 8.29 (3.9) | 0.220 | 7/21 (33.3) | 1/21 (4.8) |
| Cycle 5 6 months | 21 | 18 | 9.05 (3.3) | 0.802 | 2/18 (11.1) | 0 (0) |

* p value was calculated using paired T-test

** values show numbers with data available
